# Contrast Relative Humidity Response of Diverse Cowpea (*Vigna unguiculata* (L.) Walp.) Genotypes: Deep Study Using RNAseq Approach

**DOI:** 10.3390/ijms252011056

**Published:** 2024-10-15

**Authors:** Ekaterina A. Krylova, Marina O. Burlyaeva, Varvara E. Tvorogova, Elena K. Khlestkina

**Affiliations:** 1 N.I. Vavilov All-Russian Institute of Plant Genetic Resources, 190000 Saint Petersburg, Russia; m.burlyaeva@vir.nw.ru (M.O.B.); v.tvorogova@spbu.ru (V.E.T.); director@vir.nw.ru (E.K.K.); 2 Department of Genetics and Biotechnology, Saint Petersburg State University, 7/9 Universitetskaya Emb, 199034 Saint Petersburg, Russia; 3 Plant Biology and Biotechnology Department, Sirius University of Science and Technology, 1 Olympic Avenue, 354340 Sochi, Russia

**Keywords:** cowpea, transcriptomics, differentially expressed gene, high relative humidity

## Abstract

Cowpea (*Vigna unguiculata* (L.) Walp.) is appreciated for its suitability for cultivation and obtaining good yields in relatively extreme farming conditions. It is resistant to high temperatures and drought. Moreover, food products prepared from *Vigna* are rich in many nutrients such as proteins, amino acids, carbohydrates, minerals, fiber, vitamins, and other bioactive compounds. However, in East and Southeast Asia, where the products of this crop are in demand, the climate is characterized by excessive humidity. Under these conditions, the vast majority of cowpea varieties tend to have indeterminate growth (elongated shoot length) and are unsuitable for mechanized harvesting. The molecular mechanisms for tolerance to high relative humidity remain the least studied in comparison with those for other abiotic stress factors (drought, heat, cold, flooding, etc.). The purpose of the work was to reveal and investigate differentially expressed genes in cowpea accessions having contrasting growth habits (determinate and indeterminate) under humid and drought conditions. We performed RNA-seq analysis using selected cowpea accessions from the VIR collection. Among the genotypes used, some have significant changes in their plant architecture in response to high relative humidity, while others were tolerant to these conditions. In total, we detected 1697 upregulated and 1933 downregulated genes. The results showed that phytohormone-related genes are involved in cowpea response to high relative humidity. DEGs associated with jasmonic acid signaling are proposed to be key contributors in the maintenance of compact architecture under humid conditions.

## 1. Introduction

Cowpea (*Vigna unguiculata* (L.) Walp.) is a valuable warm-season grain legume. It is grown for young leaves, juicy vegetable pods, and dry seeds, consumed as common beans. The seeds of *V. unguiculata* are characterized by a high content of proteins, carbohydrates, macro- and microelements, low lipid content, and the presence of biologically active compounds. The vegetable (asparagus bean) pods of this crop are known for their good taste and nutritional value [1,2,3,4,5,6].

Due to the features of the attached plant lifestyle, their development and growth are coordinated with the action of various biotic (insect pests, viruses, and diseases) and abiotic stresses. All these factors have a negative influence on plant growth. Abiotic stresses include drought and flooding, high and low temperatures, salt stress. *V. unguiculata* is suitable for growing under conditions recognized as extreme for the successful production of other legumes. It is adapted to areas with high temperatures and drought, and is also grown in different soil types [7,8]. In Russia, cowpea is grown in greenhouses under a certain lighting regime without any restrictions. In open air, it is successfully cultivated in regions with different climatic conditions: the Caucasus with subtropical climate, the Caspian lowland with a dry and sharply continental climate, and the Far East region that is characterized by monsoon climate [9,10]. In the Far East, monsoon climate is characterized by maximum rainfall in the summer; during some months (more often in July and August), the relative humidity (RH) in this region exceeds 90%. Only a limited list of plants can be successfully cultivated under these climatic conditions and cowpea is one of those crops. Some positive results were obtained during the introduction of cowpea in the Primorye Territory [11]; however, under these conditions, the vast majority of cowpea varieties tends to have indeterminate growth (elongated shoot length). It is known that variation in stem elongation modifies plant architecture and so it may directly influence yield [12]. Plants with a determinate growth habit are resistant to lodging and they are characterized by the synchronicity of pod maturity. Determinacy averts the inter-twining of adjacent plants and makes intercultural operations like weeding and spraying agrochemicals easier. Cowpea cultivars with a determinate growth habit are more preferred than plants with an indeterminate growth habit. These compact plants are suitable for mechanized harvesting [13,14]. This is especially important in regions with excessive RH (Primorye Territory), where farmers are forced to cultivate cowpea on trellises.

It is known that many important features of plant growth (the type of stem growth, plant length, etc.) depend on the growing conditions. There are studies on heat and flooding stress on plant development and growth [15,16,17]. However, the effects of high RH (≥85%) have not been well studied.

The foundations for studying the patterns of variability in cultivated plants depending on ecological and geographical factors were laid by academician N.I. Vavilov [18]. The influence of geographical factors on plant height was shown. Different plants change in different ways: cereal and oilseed plants vary the least in height, and climbing legumes are more variable in plant height. It was proposed that the sum of precipitation is the main factor that influences plant height [19].

Because cowpea has high nutritional value and is a valuable component of farming systems in many areas, it is important to study the effects of high RH on cowpea growth. Accessions with different growth types were grown in artificial conditions in contrast RH (at 60% and 90%) [20]. Analysis revealed the significant influence of RH on the variability in the length and width of the first leaf. The influence of humidity on the ability of plants to form a climbing shoot was shown. The different effect of RH on the growth habit type of various accessions was revealed. In high RH, some accessions with an indeterminate growth type increased the stem length, but some accessions with a similar growth pattern did not have significant shoot elongation. Only one cultivar, “Lyanchihe”, had a stable growth type and height. These characteristics (stem growth habit (indeterminate or determinate) and plant height) were independent of RH. These results are confirmed by the results of research in the variability of morphological and phenological traits of cowpea accessions contrasted by growth habit type in different ecological and geographical conditions [12]. The stem length of most studied accessions increased significantly in response to high RH. Also, the plant architecture of some genotypes changed significantly. These changes decrease the suitability of such plants for mechanized harvesting that is especially important in monsoon conditions with excessive humidity in the Primorye Territory (Vladivostok), while only one cultivar, “Lyanchihe”, was tolerant [12].

The molecular mechanisms of plant responses to high RH are not studied enough. The aim of the current work was to reveal, at the transcriptional level, the response of cowpea accessions under high RH conditions and provide a theoretical basis for cowpea stress resistance research. Finally, we studied cowpea (*V. unguiculata*) to improve its breeding programs. The identification of the molecular mechanisms that control the stability of the growth habit type (plant architecture), stem length, and its interrelationships with other morphological traits in high RH conditions will allow for intensifying more efficient breeding programs of new cowpea varieties.

## 2. Results

### 2.1. Transcriptome Sequencing and Identification of Differentially Expressed Genes

A total of 24 mRNA libraries were sequenced that were derived from the leaves of four cowpea accessions (k-6, k-642, k-1783, and k-2056) in two relative humidity conditions (three biological replicates for each accession in each condition). The Illumina NovaSeq 6000 SP high-throughput whole-genome sequencing system was used for analysis. In total, 1,016,216,189 single-end 100 bp raw reads were obtained. The number of raw reads was in the range from 33,505,812 (for k642_control_1) to 51,121,766 (for k642_control_2). The quality control of the raw reads showed their high quality, with a small number of adapter sequences. After filtering, the reads by length, quality, and after removing adapter sequences, 976,508,096 (96%) reads were retained. The percentage of clean reads ranged from 91.9% to 96.9%. On average, 89.3% reads were uniquely mapped reads to the reference cowpea genome from the Phytozome database (Vunguiculata_540_v1.2) using the STAR v.2.7.10b software (Table S3). Only one replicate of k642_control had 26% uniquely mapped reads. Of the 31,948 standard genes used for analysis, 24,350 had non-zero expression values. Genes with expression differences with a false discovery rate (FDR) < 0.05 and log base 2-transformed fold change (|logFC|) > 1 were acknowledged as differentially expressed genes (DEG) (Table S4).

**Figure 1:**
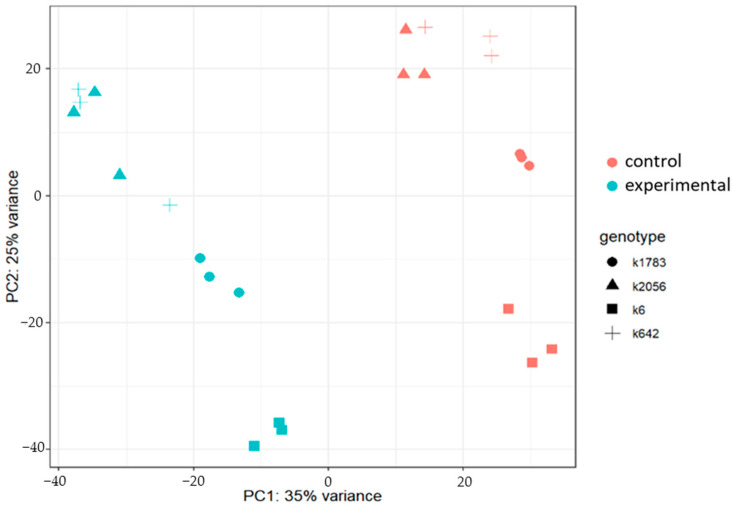
Principal component analysis (PCA) plot of all expressed genes in the RNA–seq data. The *X*-axis indicates the first principal component; the *Y*-axis indicates the second principal component. The percentage of variance explained by each PC is shown in each case. Rose color—control group (the relative humidity was equal to 60%), blue color—experimental group (the relative humidity—90%). Analysis was performed with the DESeq2 package version 1.38.3.

The principal component analysis (PCA) demonstrated a high level of differences between accessions in different relative humidity conditions (Figure 1). The percentage of the explained dispersion for the PCA model was 60% (35% for PC1 and 25% for PC2). There was clusterization of three replicates for all accessions.

**Figure 2:**
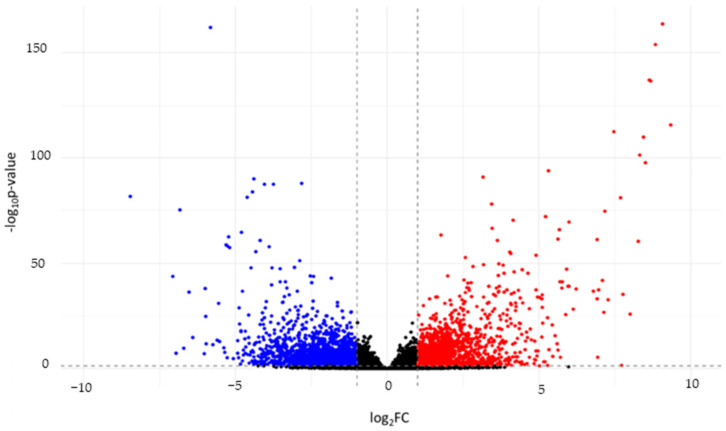
Volcano plot representing 24,350 differentially expressed genes. The *X*-axis indicates the log2-transformed gene expression fold changes between control group and experimental group of cowpea accessions. The *Y*-axis indicates the log10-transformed *p*-value. Dashed lines indicate log_2_FC and *p*-value thresholds. The scattered points represent each gene. Significant differentially upregulated genes are highlighted in red, significant differentially downregulated genes are highlighted in blue. Genes with a nonsignificant log_2_FC value and nonsignificant *p*-value are highlighted in black.

The gene expression profiles of all cowpea accessions were compared between different relative humidity conditions (experimental (high RH) versus control (low RH)). The distribution of differentially expressed genes (DEGs) by FDR and *p*-value is shown as a volcano plot. Among the DEGs, we observed a two-fold increase in the expression of 1697 genes. Two-fold decrease of expression was detected for 1933 genes (|logFC| > 1). Genes with significant expression changes are marked in the plot with red (upregulated genes) and blue (downregulated genes) dots (Figure 2).

**Figure 3:**
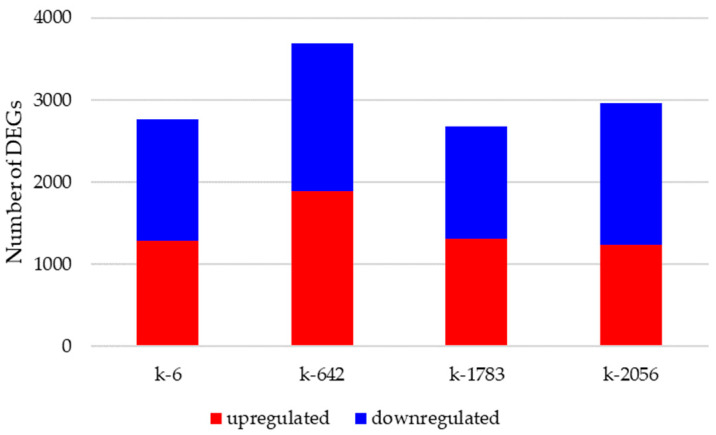
Number of DEGs identified in a comparison between the control and experimental groups for each cowpea accession.

Then, differential expression was calculated for each accession in different relative humidity conditions (control and experimental), in a total of four comparison group. The number of upregulated and downregulated genes for k6 in the control and experimental groups were 1284 and 1482, respectively, for k642, it was 1886 upregulated and 1803 downregulated genes, for k1783 it was 1306 upregulated and 1374 downregulated genes, and for k-2056, it was 1242 upregulated and 1715 downregulated genes (Figure 3). So, in total, the number of DEGs identified for k-642 was the highest in comparison with the DEG number for other accessions.

**Figure 4:**
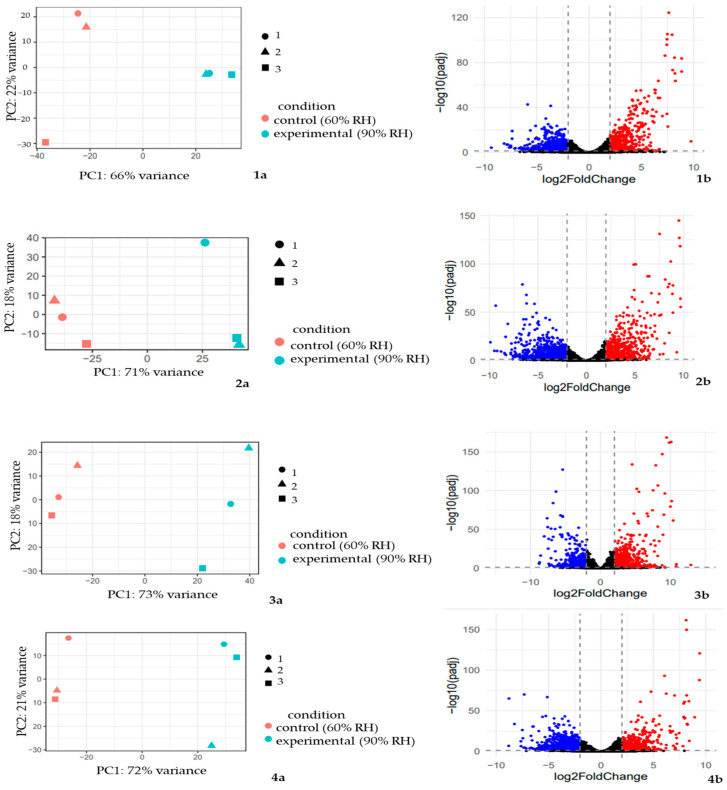
Principal component analysis (PCA) plots and volcano plots of expressed genes in the RNA-seq data for four comparisons. (**1a**,**1b**) PCA plot and volcano plot for k6; (**2a**,**2b**) PCA plot and volcano plot for k642; (**3a**,**3b**) PCA plot and volcano plot for k1783; (**4a**,**4b**) PCA plot and volcano plot for k2056. The *X*-axis on the PCA plots indicates the first principal component; the *Y*-axis indicates the second principal component. The percentage of variance explained by each PC is shown in each case. Rose color—control group (the relative humidity was equal to 60%), blue color—experimental group (the relative humidity—90%). Analysis was performed with the DESeq2 package version 1.38.3. The *X*-axis on the volcano plots indicates the log2-transformed gene expression fold changes between the control group and experimental group of cowpea accession. The *Y*-axis indicates the log10-transformed *p*-value. Dashed lines indicate log_2_FC and *p*-value thresholds. The scattered points represent each gene. Significantly differentially upregulated genes are highlighted in red, and significantly differentially downregulated genes are high-lighted in blue. Genes with a nonsignificant log_2_FC value and nonsignificant *p*-value are highlighted in black.

The principal component analysis (PCA) demonstrated a high level of differences between the plants of each accession in different relative humidity conditions (Figure 4). The percentage of the explained dispersion for the PCA model ranged from 88% to 93% (66–73% for PC1 and 18–22% for PC2) (Figure 4(1a,2a,3a,4a)). The volcano plots show the distribution of differentially expressed genes by FDR and *p*-value. Among them, we observed a two-fold increase in expression and a two-fold decrease in expression in each comparison group of the gene expression of four cowpea accessions that were grown at 60 and 90% RH. Genes with significant expression changes are marked in the diagram with red (upregulated genes) and blue (downregulated genes) dots (Figure 4(1b,2b,3b,4b)).

**Figure 5:**
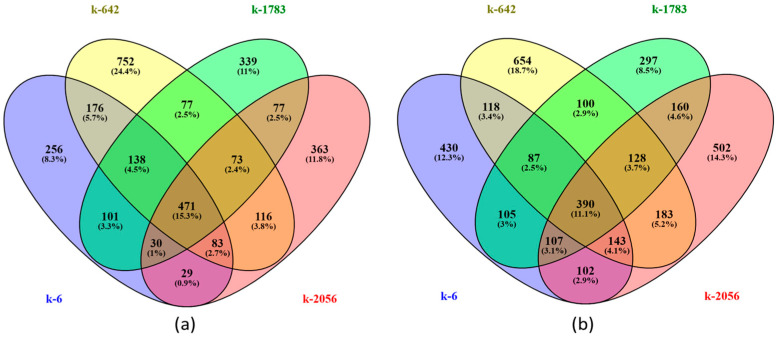
Venn diagrams representing the overlap between DEGs identified in four cowpea accessions in the control and experimental groups (two relative humidity conditions): (**a**) upregulated genes and (**b**) downregulated genes. Accessions are marked by different colors in letters.

In a comparison of the numbers of DEGs between accessions, there were 471 upregulated and 390 downregulated genes between all studied accessions (Figure 5). There were 256 upregulated genes specific for k6, 363 genes for k2056, and 339 genes for k1783. The number of k-642-specific upregulated genes was almost twice as much as the number of common genes for the four studied genotypes; there were 752 genes (Figure 5a). There were 430 downregulated genes specific for k6, 502 genes for k2056, and 654 genes for k642 (Figure 5b). The number of k-1783-specific downregulated genes (297 genes) was less than the common genes for the four studied genotypes.

### 2.2. Gene Ontology Term Enrichment in Lists of Differentially Expressed Genes

**Figure 6:**
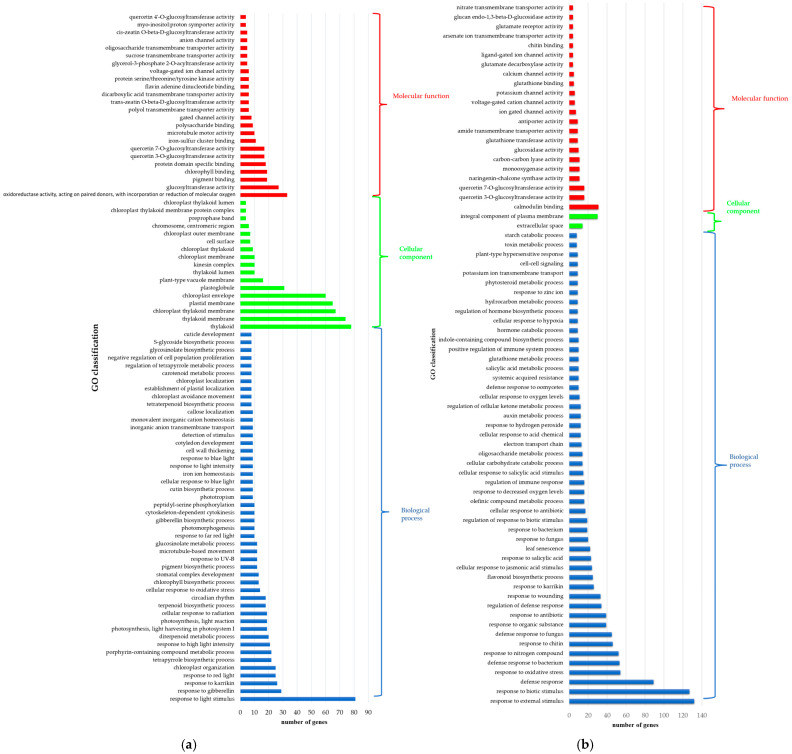
GO enrichment analysis of the (**a**) upregulated genes and (**b**) downregulated genes identified in four cowpea accessions in the control and experimental groups (two relative humidity conditions). For the BP categories, the top 50 GO terms are presented.

To functionally characterize the DEGs, GO enrichment analysis of each group was conducted. The lists of DEGs of the cowpea accessions compared between different relative humidity conditions (high RH versus low RH) were annotated by the enrichment analysis of the GO terms, and GO functions were in terms of molecular function (MF), biological processes (BPs), and cellular components (CCs) (Table S5). As shown in Figure 6, among the upregulated genes, enriched pathways were mainly involved in response to light stimulus (BP), plastid, and thylakoid membranes (CC). In the MF category, DEGs were involved in enzyme activities (oxidoreductase and glucosyltransferase activity) and pigment binding. In high RH, downregulated genes were mainly enriched in terms of response to external and biotic cellular stimulus (BP), anatomical entity (CC), and calmodulin binding (MF).

In addition, GO enrichment analysis of each accession (accession in high RH versus the same accession in low RH) was conducted. Grain accession “Clay” (k-6) with an indeterminate growth habit type was mainly enriched in terms of response to abiotic stimulus, response to light stimulus, pigment metabolic process, response to external stimulus, response to biotic stimulus, and organelle and chloroplast envelope, organelle subcompartment, integral component of the plasma membrane, and protein-domain-specific binding, pigment and chlorophyll binding, calmodulin binding, glucosyltransferase activity in the BP, CC, and MF categories, respectively.

Among the top GO terms of the DEGs, vegetable accession k-642 with an indeterminate growth habit type was mainly enriched by biological processed related to response to light stimulus, response to nitrogen compound, response to chitin, defense response to fungus, lipid biosynthetic process, pigment metabolic process, isoprenoid metabolic process, and secondary metabolite biosynthetic process. In the CC category, there were organelles and the chloroplast envelope, organelle subcompartment, thylakoid membrane, and ribosomal subunit. In the top list of the DEGs in MF, the GO terms “pigment binding” “chlorophyll binding”, “protein domain specific binding”, “structural constituent of ribosome” were found for k-642 in high RH conditions.

Grain accession k-1783 with an indeterminate growth habit type was mainly enriched in terms of response to light stimulus, pigment metabolic process, isoprenoid metabolic process, dephosphorylation, cellular response to abiotic stimulus, cellular response to fatty acid, and organelle envelope, thylakoid membrane, and carbohydrate binding, glucosyltransferase activity, pigment and chlorophyll binding in the BP, CC, and MF categories, respectively.

The top GO terms of the DEGs (from BP) of k-2056 (vegetable cultivar Lyanchihe) were mainly related to response to light stimulus, photosynthesis, light reaction, pigment metabolic process, isoprenoid metabolic process, dephosphorylation, response to external stimulus, response to biotic stimulus. The lists of DEGs were found to be enriched with GO terms related to the organelle envelope, thylakoid membrane, anchored component of plasma membrane, and oxidoreductase activity, phosphatase activity, pigment and chlorophyll binding, calmodulin binding, glucosyltransferase activity, and transmembrane receptor protein serine/threonine kinase activity.

Thus, following the comparison of each accession in two RH conditions, the top GO terms of the DEGs were mainly enriched in terms of response to abiotic and light stimulus, organelle and chloroplast envelope, chlorophyll binding, and glucosyltransferase activity.

Common upregulated genes (471 genes) had 49 enriched GO terms in the BP category, with many of the DEGs associated with response to abiotic stimulus and response to light stimulus. We identified genes involved in photoperiodism and flowering (GO:0048573), among which are *Vigun05g024400* (*AT5G15850*, that encodes the protein CONSTANS that has a crucial role in flowering regulation), *Vigun09g004100,* and *Vigun10g153300*, that encode a MYB-related putative transcription factor involved in the circadian rhythm, *Vigun07g078900* (MYB-like transcription factor), and *Vigun08g171500* (E3 ubiquitin–protein ligase COP1). In addition, upregulated genes had 10 enriched GO terms in the MF category and the DEGs were mainly associated with oxidoreductase activity.

The common downregulated genes (390 genes) had 14 enriched GO terms in the BP category, with most of the DEGs related to biological processes involved in interspecies interaction between organisms, response to external biotic stimulus, and defense response to other organisms. Also, genes were identified that related to the regulation of flower development (GO:0009909), among which were *Vigun03g011300* (*AT1G68050*, that encodes FKF1 and regulates transition to flowering) and *Vigun01g227200* (*AT2G40080* encodes ELF4). In the MF category, the GO terms of common downregulated genes were enriched by eight terms, mainly genes related to cation transmembrane transporter activity and oxidoreductase activity.

Also, in the list of upregulated genes, GO terms “response to ethylene” (GO:0009723) and “response to gibberellin” (GO:0009739) were found. Among downregulated genes were “response to ethylene” (GO:0009723) and “auxin metabolic process” (GO:0009850).

### 2.3. Changes in Phytohormone Signaling Pathway-Related Gene Expression in High RH Conditions

Among the lists common for all accessions of upregulated and downregulated genes, genes relating to ethylene (ET)- and gibberellin (GB)-, and ET- and auxin-associated pathways, respectively, were identified. Changes in other phytohormone signaling pathway-related gene expression were not observed.

**Figure 7:**
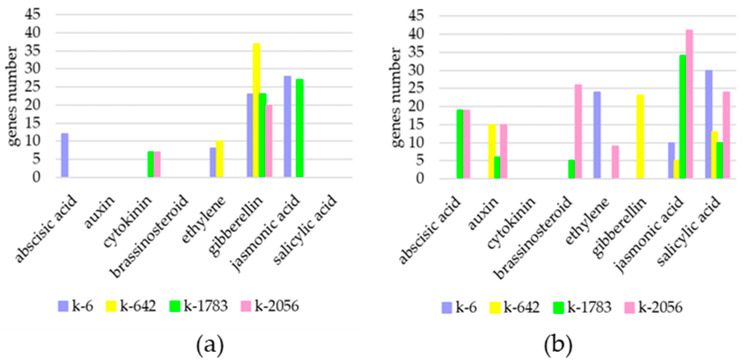
Number of DEGs associated with plant hormone biosynthesis, metabolism, and signal transduction pathways. Genes identified in four cowpea accessions in the control and experimental groups (two relative humidity conditions): (**a**) upregulated genes and (**b**) downregulated genes.

Analysis of lists of individual DEGs for each accession was carried out. Differential changes were revealed for plant hormone (abscisic acid (ABA), auxin, brassinosteroid (BR), cytokinin (CT), ET, GA, jasmonic acid (JA) and salicylic acid (SA)) biosynthesis, metabolism, and signal transduction pathways in all the studied accessions (Figure 7).

For all accessions, GA-related expression increase was identified; at the same time, most of these genes were in k-642 in high RH conditions. Gene expression related to auxin-, BR-, and SA-related pathways was not increased. CT-related genes were upregulated only in k-1783 and k-2056, ET-related genes only in k-6 and k-642, and JA-related pathways only in k-6 and k-1783. ABA-related upregulated genes were identified only in k-6.

On the other hand, in high RH, there were more plant hormone-related genes with an expression decrease. Among downregulated genes, only CT-related genes were not found. On the contrary, genes that related to the auxin and BR pathways decreased expression, and this was not observed among upregulated genes. In addition, for all accessions, downregulated genes associated with SA pathways were identified. JA-associated pathway genes were observed in all accessions, but it should be noted that most of these genes were for k-2056, and less for k-642.

The most pronounced changes in the expression level of phytohormone-related genes were observed in k-2056 (cultivar Lyanchihe). In total, 161 genes (up- and downregulated genes) were identified for this accession. Despite the fact that substantially greater DEGs were identified for k-642 than for other genotypes, the number of hormone pathway-related genes (in total, 103 genes) was lower for this accession than for others (Figure 7).

We analyzed JA-related genes that were only genotype-specific. In k-2056, some JA-related genes were downregulated. On the list of unique downregulated genes in GO:2000022 (regulation of jasmonic acid mediated signaling pathway), we identified three genes coding for the suppressor proteins jasmonate ZIM domain-containing protein (JAZ-protein): *Vigun03g399500*, *Vigun10g161900*, and *Vigun02g160700*. Also, *Vigun01g228500*, *Vigun10g080400*, and *Vigun08g027900* decreased expression. In addition, we found unique downregulated genes in GO: 0071395 (cellular response to jasmonic acid stimulus).

In GO:0071395 (cellular response to jasmonic acid stimulus), ethylene response factor 1 (Vigun07g178200), cytochrome P450 CYP2 subfamily protein (Vigun06g026800), and GRX480 from glutaredoxin family (Vigun11g098600) were downregulated in high RH only in k-2056.

Based on the results of the transcript analysis, we suggest that cowpea accession k-2056 (cultivar Lyanchihe) responds to high RH conditions primarily by regulating hormone signaling, with the most pronounced changes occurring in JA-related pathways.

### 2.4. Quantitative Reverse-Transcription PCR Verification of Gene Expression

**Figure 8:**
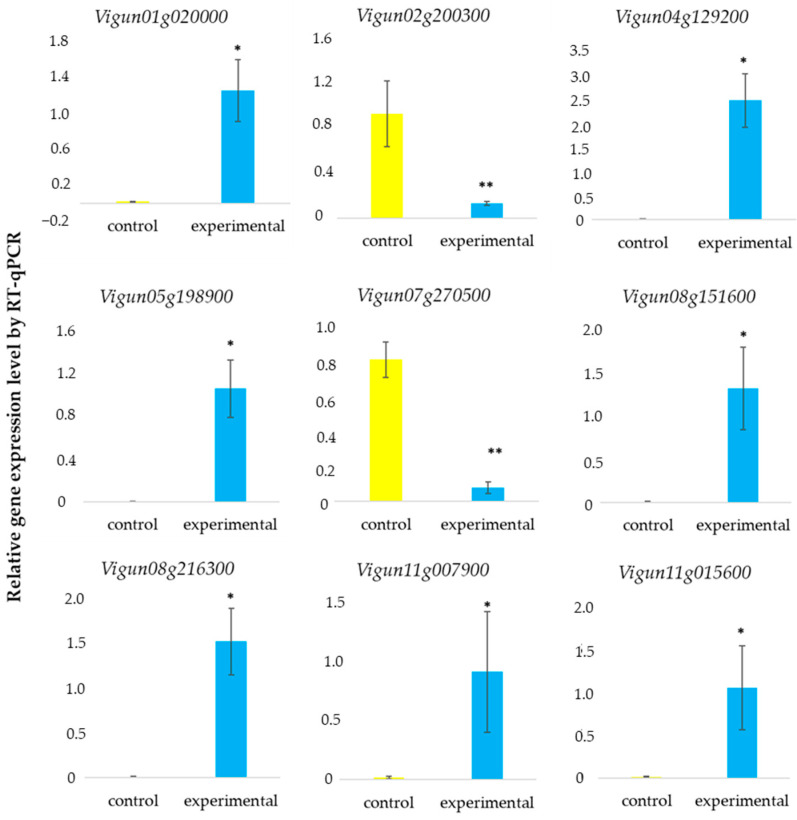
qRT-PCR validation of nine DEGs for k-2056 in high RH (experimental) versus low RH (control). Data were normalized to the expression of *VuUBQ10* (*Vigun07g244400*) encoding ubiquitin. Each sample was amplified in three technical replicates. Significant differences between the mean values are indicated (* *p* ≤ 0.001, ** *p* ≤ 0.05) (*t*-test).

The expression of nine genes was confirmed by qRT-PCR. These genes code for tryptophan synthase beta chain (Vigun02g200300), chlorophyll a/b binding protein (Vigun04g129200), light-harvesting complex II chlorophyll a/b binding protein 3 (Vigun05g198900), protein with a B-box zinc finger (Vigun07g270500), regulator of chromosome condensation (Vigun08g151600), and light-harvesting complex II chlorophyll a/b binding protein 2 (Vigun08g216300). The expression of *Vigun01g020000*, *Vigun11g007900*, and *Vigun11g015600* genes was also analyzed. The relative expression levels (in vitro data) obtained from qRT-PCR exhibited trends similar to those of the log_2_FC from RNA-seq (in silico data), thus indicating the reliability of our RNA-seq data (Figure 8, Table 1).

## 3. Discussion

It is known that plant morphology is affected by relative humidity. Shoot elongation, increased leaf size (leaf length and width), and internode length are caused by high RH [21,22,23,24]. Besides that, some plants are characterized by enhanced dry weight at a high RH. During the first two weeks of growth in climatic chambers at an RH level of 95%, an increased dry weight of the leaves, stems, and roots was shown. The leaf area on the main and lateral stems was also increased. At 6 weeks, there was no effect of high RH on plant height, number of leaves on lateral shoots, and number and length of lateral shoots [22]. The number of flowers and leaves, biomass, and flowering time differed non-significantly between plants grown at a high and moderate RH [25,26].

At a high RH, most plants had an increased density and size of the stomata [27]. A universal response to a high RH during leaf expansion is the formation of bigger stomata with larger pores. Stomatal density depends on plant species and it is specific for different leaf sides [25,26,27,28]. Stomata close in response to a low RH. It is a very important issue of plant responses to avoid water loss. A decreased vein density at a high RH was shown. Guard cells are sensitive to environmental signals such as temperature, light, water and RH, and phytohormone levels.

Our previous study of cowpea accessions with different growth habits in controlled artificial conditions showed that the variability in the length and width of the first leaf depends on the RH. The variation in plant length was due to a complex of factors. This trait was interrelated with growth conditions and genotypic characteristics. In addition, the different effect of RH on the growth habit type of various accessions was revealed. Therefore, for some accessions, the influence of RH on the ability of plants to form a climbing shoot was shown [20]. This finding corresponded to the results of the study in field conditions in contrast RH conditions (in the Caucasus, the Caspian lowland, and the Far East region) [9,12]. It was shown that cowpea genotypes differed by response to a high RH. Significant shoot elongation was observed for most studied accessions. In addition, in the monsoon climate of Primorye Territory, some genotypes changed growth habit type from determinate to indeterminate. Plants of the cultivar Lyanchihe had a stable architecture. Non-significant shoot elongation in high RH conditions was shown for k-2056 (cultivar Lyanchihe) only [12]. In the current study, on the basis of the investigation results of variability in morphological traits in artificial and different ecological and geographical conditions, four genotypes were chosen as the model for transcriptome analysis. These accessions had different growth habit types.

A high RH also affects phytohormone levels. Hence, the concentration of abscisic acid decreased at a high RH, while the ethylene level increases [29,30,31,32,33]. Upon combined transcriptome and metabolome analyses, differentially expressed genes (DEGs) and differentially accumulated metabolites in response to a high RH were identified [34]. It was supposed that abscisic acid-, auxin-, and jasmonic acid-related genes may be involved in the response to high-RH stress in quinoa seedlings [34].

On the basis of the transcriptome analysis results, we identified DEGs encoding transcription factors. These differentially expressed transcription factors belonged to different families, such as AP2/ERF, GATA, MYB, NAC, WRKY, and B3. Most of the transcription factors belonged to the WRKY and AP2/ERF transcription factor families. They were downregulated in plants in high RH compared to the plants in low RH conditions. The genes encoding transcription factor B3 family (auxin response factors) were upregulated. It is known that AP2/ERF, WRKY, and B3 genes are to be associated with stress responses in plants. We suppose that changes in the expression level of these transcription factors, particularly those related to phytohormone signaling, are among the important mechanisms underlying the response to high RH in cowpea. Also, we found that plant hormone signaling pathways were significantly altered in response to high RH stress. For all accessions, DEGs related to ET- and GB-, and ET- and auxin-associated pathways were identified. Among the lists of individual DEGs, most GA-related upregulated genes were in k-642 in high RH conditions. Noteworthily, this accession was characterized by significant (more than twice) shoot elongation in high RH in the Far East region [12]. Other hormone-related genes (CT, ET, JA, ABA) were not found for all accessions.

In high RH, more plant hormone-related genes with an expression decrease were observed. Genes related to the auxin and BR pathways were identified among the list of individual downregulated genes of some accessions, and only CT-related genes were not found. It is important that only SA- and JA-related genes were found for all studied accessions.

The plant hormone signaling pathways in k-2056 (cultivar Lyanchihe) in response to high RH stress were altered the most significantly. Namely, JA-associated pathway genes were more represented than other hormone-related genes. We found nine downregulated genes that participate in the jasmonic acid-mediated signaling pathway (GO:2000022) and the cellular response to jasmonic acid stimulus (GO:0071395) for this accession only. On the gene list, suppressor protein jasmonate ZIM domain-containing protein (JAZ-protein) that encode *Vigun03g399500* (JAZ1), *Vigun10g161900* (JAZ6), and *Vigun02g160700* (JAZ2) were found. In addition, in high RH, *Vigun01g228500* and *Vigun10g080400* encoding jasmonate-induced oxygenases were downregulated in k-2056. They are homologs of *JOX2* (*AT5G05600*) and *JOX1* (*AT3G11180*), respectively. These proteins hydroxylate jasmonic acid to form inactive 12-OH-JA [35]. *Vigun08g027900* (*MYB73*), *Vigun06g026800* (*CYP71B7*), and *Vigun11g098600* (*GRX480*) expression decreased. Also, on the list of unique downregulated genes, we found *Vigun07g178200*, which encodes ERF1.

It is known that JA and its derivatives (jasmonates JAs) regulate plant growth and development. Initially, it was identified as a stress-related hormone in higher plants. JAs are involved in a variety of processes, for example, flower development, leaf senescence, inhibition of petal expansion, inhibition of root and hypocotyl growth, and stomatal opening. Jasmonate inhibits the transition from vegetative to reproductive maturity in *Arabidopsis* [36,37].

Under normal conditions, the level of JAs in the cytoplasm is very low and the genes involved in JA synthesis are inactive. In this state, jasmonate ZIM domain-containing proteins (JAZ-proteins) recruit NOVEL INTERACTOR OF JAZ (NINJA; an adaptor protein) and TOPLESS (TPL; a co-repressor) to repress various downstream transcription factors (TFs) via direct protein interactions. Plant flowering is controlled by a gene network in which *TOE1* and *TOE2* are presented. It was shown that flowering is inhibited by the interaction of COI1 and JAZ, and *TOE1* and *TOE2* binding with JAZ limits flowering via the inhibition of transcription *FLOWERING LOCUS T* [38]. In our study, *Vigun05g252100* (*TOE1*) was downregulated only in k-6 and k-642 in high RH. These two accessions are characterized by an indeterminate growth habit type. Moreover, in monsoon climate, significant shoot elongation and, accordingly, a later transition to flowering, were observed.

Therefore, JAZ proteins play a pivotal role in a JA signaling process and are widely implicated in regulating plant development processes [39]. It was shown that overexpressing JAZ1-like with Jas-domain deletion in *Chrysanthemum morifolium* resulted in late flowering. In our study, a decrease in *Vigun03g399500* (a homolog of *AtJAZ1*) was identified only in one accession: k-2056. This accession was characterized by stable plant architecture, independent of RH conditions. This gene determines shoot growth habit and the terminal meristem switching from a vegetative to a reproductive state to produce a terminal flower. On the basis of previous data, it is supposed that decrease in *JAZ* expression probably activates flowering.

Noteworthily, among unique downregulated DEGs (for k-2056), we identified *JOX1* and *JOX2*. This is consistent with the literature data. It was shown that the mutants of these genes had an increased JA level and were characterized by reduced growth [35].

Interactions among plant hormone signals are very important for plant responses to biotic and abiotic factors. JA does not have an independent regulatory role. There are interactions between JA and other hormone pathways in response to abiotic stresses. It is known that JA inhibits stem growth in *Nicotiana attenuate* [40]. The high JA levels suppressed stem elongation and the accumulation of secondary metabolites. GA biosynthesis and the transcription of GA20ox and possibly GA13ox, the key genes in GA production, were strongly inhibited by JA. It was shown that plants with defects in JA production or signaling have problems with defense, but they have normal vegetative growth [40].

## 4. Materials and Methods

### 4.1. Materials

Four genotypes of *V. unguiculata* from the collection of the N.I. Vavilov All-Russian Institute of Plant Genetic Resources (VIR) were used in this study. The experimental accessions were characterized by different types of stem growth habit types and also reflected the entire spectrum of variability of this trait in various climatic conditions. The range of morphological variability (stem length) of the selected accessions in three ecological and geographical conditions at VIR experimental stations is presented in Figure S1 [12]. The accessions were selected based on the research results of the variability in morphological traits in controlled (artificial) and field conditions that are contrasted by RH [12,20,41]. A stable architecture of the cultivar “Lyanchihe” independent of climatic conditions (in the Astrakhan Province (sharply continental climate), Primorye Territory (monsoon climate), and Krasnodar Territory (subtropical climate)) was observed. In the years during which we analyzed morphological variability in the Astrakhan Province, the average sum of active temperatures above 15 °C was 3549 °C and varied from 3460 °C to 3785 °C. In Krasnodar Territory, the average sum of the active temperatures was 3146 °C and varied in 3460 °C to 3785 °C range. While in Primorye Territory, it equaled to 1841 °C and varied from 3146 °C to 3354 °C. The average sum of precipitation in the Astrakhan Province was 223 mm (197–250 mm), in Krasnodar Territory, it was 678 mm (280–968 mm), and in Primorye Territory, it was 754 mm (462–1035 mm). Relative humidity during the period of plant vegetation in the Astrakhan Province varied from 45% to 59%. In Krasnodar Territory, it ranged from 74% to 81%, while in Primorye Territory, relative humidity varied from 63% to 94% range. In particular, high RH occurs in June, July, and August, when active plant growth is observed (Figure S2). Excessive humidity and precipitation had a minimal effect only on the cultivar “Lyanchihe”; the plants retained dwarfness. For k-642, we observed greater stem elongation and changes in growth habit type to indeterminate [12]. In the section below, there is a description of accessions studied in this research.

Accession k-6 (*V. unguiculata* subsp. *unguiculata* (L.) Walp., grain cultivar “Clay”) was obtained from the extract in 1921 in the USA. It was grown on seeds and green mass. It was of the late maturing variety. The growing season from germination to seed maturation was 117–120 days. Plants have an indeterminate growth habit type. The stem length reaches 210 cm in the Astrakhan Province, and in the Primorye Territory, it is about 250 cm [12]. The powerful shrub has a large number of branches (3–6) and leaves. Green pods in the stage of technical ripeness are wide and 15–20 cm in length; they have the parchment layer and fibers. The 1000-seed weight is 137–140 g. The seeds are light beige. The potential yield of the seeds is 2.5–2.6 t/ha.

Landrace k-642 (*V. unguiculata* subsp. *sesquipedalis* (L.) Verdc., vegetable accession) was collected by N.I.Vavilov during the 1929 expedition to China. It is grown on the production of vegetable pods with high nutritional qualities. Pods are used in cooking, canning, and freezing. They are an early maturing accession. The growing season from germination to technical pod ripeness is 50–52 days, until seed maturation, at 65–68 days. It is a semi-shrub accession with a curly tip. Stem length is 70–110 cm in the Astrakhan Province, while in the Far East monsoon climate, the stem significantly elongates and reaches 350–380 cm, and plants have an indeterminate growth habit type [12]. The number of branches is 1–5. Pods in the stage of technical ripeness are wide, light green, 42–50 cm in length, and without parchment layer and fibers. The potential yield of pods at the stage of technical ripeness is 16.1–20.1 t/ha, seeds—1.9–2.4 t/ha. The 100-pods weight is 1180–1360 g, and the 1000-seed weight is 130–140 g. The seeds are red-brown.

Accession k-1783 (*V. unguiculata* subsp. *cylindrica* (L.) Verdc.) was obtained in 1985 from Germany. It was grown on seeds and green mass. It is a medium maturing accession; the growing season from germination to seed maturation is 75–80 days. Plants are erect with the curly tip and they have indeterminate growth habit type. The stem length is 50–80 cm in the Astrakhan Province, and in the Primorye Territory, the stem reaches 260 cm [12]. The number of branches is 1–4. The pods are narrow and short (10–12 cm in length); in the stage of technical ripeness, they are yellow and are directed upward. Pods have a parchment layer and fibers. The potential yield of seeds is 2.0–2.1 t/ha. The 1000-seed weight is 50–60 g. The seed shape is cylindrical and the seed color is cream.

Cultivar k-2056 (*V. unguiculata* subsp. *sesquipedalis*, modern vegetable cultivar “Lyanchihe”) was developed in Russia in the Primorye Territory as a result of the selection from accessions of Chinese origin. It is grown on vegetable pods with high nutritional qualities. Pods are used in cooking, canning, and freezing. It is an early maturity cultivar and the growing season to seed maturation is 57–65 days. The cultivar has a compact bush shape, with a determinate growth habit type. The stem length is 15–50 cm in the Astrakhan Province and 30–100 cm in the Primorye Territory. The number of branches per plant is 1–3. Pods in the stage of technical ripeness are narrow, cherry-colored, 20.0–30.0 cm in length, and do not contain a parchment layer and fibers. The potential yield of pods at the stage of technical ripeness is 21.0–24.0 t/ha, and of seeds, 2.3–2.6 t/ha. The 100-pod weight is 1400–1420 g and 1000-seed weight is 120–125 g. The seeds are red–brown.

Uniform, full, and intact seeds were selected from each of the 4 accessions. The seeds were seeded in round seedling pots (80 × 80 mm), where the sowing depth was 2 cm. Seeds were planted in universal nutrient soil (Terra Vita, Russia). The indicators of the content of the main nutrients in the soil were as follows: nitrogen (NH_4_ + NO_3_), at least 150 mg/L; phosphorus (P_2_O_5_), at least 270 mg/L; potassium (K_2_O), at least 300 mg/L; the pH of the salt suspension, 6.0–6.5. Additional treatments at the stage of germination and the vegetation period of the plants were not used.

**Figure 9:**
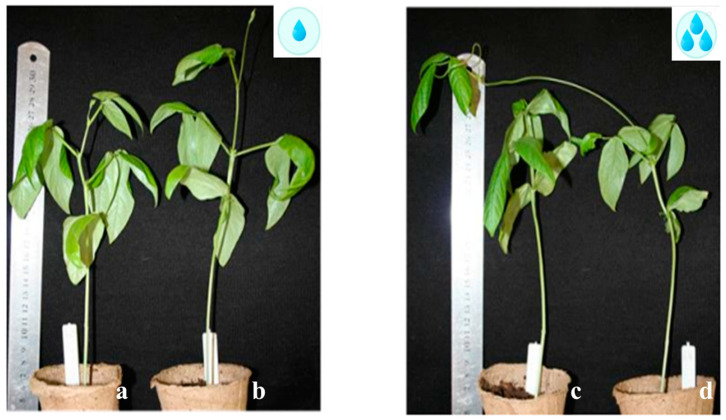
Cowpea accessions k-2056 (**a**,**c**) and k-642 (**b**,**d**) in two climatic chambers: (**a**,**b**) 60% RH (control group), (**c**,**d**) 90% RH (experimental group). The formation of climbing shoot for k-642 in high RH was observed (**d**), and for k-2056, there was no formation of such shoots (**c**).

Plants were grown under controlled artificial conditions in two climatic chambers (Voltech LLC, Volgograd, Russia) at a 12 h light day at a constant temperature (25 °C) and at a light intensity of 3000 Lx. The plants were divided into two experimental groups. In the first experiment, the relative humidity was equal to 60% (control group). In the second, 90% (experimental group). Thus, each accession was grown in contrasting humidity conditions (Figure 9). In 10–14 day plants, the first leaves (when they were fully opened) of the experimental and control groups of each accession were sampled separately, snap-frozen in liquid nitrogen, and stored at −80 °C. Three biological replicates were included in this study.

### 4.2. Isolation of Total RNA

The first leaves were frozen in liquid nitrogen and homogenized with a pestle in a mortar. Total RNA was extracted in three biological replicates using the RNeasy Plant Mini Kit (Qiagen, Hilden, Germany), according to the manufacturer’s instructions (www.qiagen.com, accessed on 20 November 2021). DNase treatment with the RNase-free DNase set (Qiagen, Hilden, Germany) was used for the removal of DNA. The quality of the isolated RNA was evaluated in 1% agarose gel prepared based on the TAE buffer with the addition of ethidium bromide as an intercalating dye. The Sky-High 250 b–10 kb marker (BioLabMix, Novosibirsk, Russia) was used as a DNA molecular weight marker. The resulting isolation products were visualized using the BioRad ChemiDoc MP gel-documenting system (Bio-Rad Laboratories, Moscow, Russia). Concentrations of the isolated RNA were measured using a NanoDrop™ 2000/2000c spectrophotometer (Thermo Fisher Scientific Inc., Waltham, MA, USA).

### 4.3. RNA Library Preparation

The TruSeq mRNA-stranded reagent kit (Illumina, San Diego, CA, USA) was employed to enrich total RNA samples with the poly(A+) fraction. cDNA synthesis was carried out using Superscript II Reverse Transcriptase, followed by second-strand cDNA synthesis. The obtained cDNA was used to prepare libraries compatible with Illumina sequencing technology. The quality of the obtained libraries was checked using the Fragment Analyzer (Agilent, Moscow, Russia). Quantitative analysis was performed by a real-time polymerase chain reaction (qPCR). After quality control and cDNA quantity evaluation, the library pool was sequenced using the Illumina NovaSeq 6000 SP high-throughput whole-genome sequencing system (www.illumina.com, accessed on 18 December 2021). The volume of each library was 30 uL, and the concentration was in the range from 3 ng/μL to 10 ng/μL. The libraries were pooled in an equimolar ratio in order to get an equal sequencing depth. The volume of each pooled library was calculated based on its concentration. There were 24 barcoded libraries (3 biological replicates × 4 cowpea accessions × 2 RH conditions). The sequenced reads were deposited in the NCBI Sequence read archive (BioProject ID: PRJNA1169611; SAMN44078771–SAMN44078794).

### 4.4. RNA-Seq Data Analysis

The quality of single-end 100 bp raw reads was checked using FASTQC v.0.12.1 [42]. The filtering of the libraries was performed using fastp software v.0.23.2 with the following parameters: “-l 50 --cut_front --trim_front1=20 --cut_right” [43]. It resulted in removing unidentified bases (N) or bases with a Phred quality score below 20 from both 3′ and 5′ -ends of the read, and removing the reads with a length less than 50. Then, the quality of the filtered reads was reevaluated. The MultiQC v.1.10.1 was used for combining the quality control reports [44].

The filtered reads were then mapped onto the *Vigna unguiculata* (v1.2) reference genome from the Phytozome database (http://phytozome.jgi.doe.gov/ (accessed on 2 September 2023)). The STAR v.2.7.10b tool with default parameters was applied to map the short-read libraries to the reference cowpea genome [45]. The number of reads aligned to each gene was counted with the help of the featureCounts function in the Subread software package v.2.0.1 [46]. The raw read count data are presented in Table S1.

Differential gene expression analysis was performed using the DESeq2 package version 1.38.3 (Wald test was applied) [47]. Raw reads were subjected to DESeq2. Genes with a low expression (which normalized read counts were lower than 1) were eliminated. Genes expression after normalization are presented in Table S2. Differential expression between the groups of samples was detected via the generalized linear model approach. Differential expression was calculated between each experimental group in different conditions (one accession in low RH versus the same accession in high RH). Thus, 4 comparisons were performed, namely comparison of the gene expression of 4 cowpea accessions that were grown at 60 and 90% RH. Genes with a false discovery rate (FDR) of <0.05 and log base 2-transformed fold change (|log2(FC)|) > 1 were considered differentially expressed. A volcano plot showing the distribution of differentially expressed genes (DEGs) was made using the EnhancedVolcano package [48].

For the functional annotation of differentially expressed genes (DEGs), Gene Ontology (GO) enrichment analysis and metabolic pathway involvement analysis were undertaken. The enriched Gene Ontology groups were analyzed using the GSEAbase package version 1.64.0. For each comparison performed for the differential expression analysis, the lists of upregulated and downregulated genes were examined separately. GO terms with an adjusted *p*-value < 0.05 were regarded as significantly enriched.

### 4.5. Quantitative Reverse-Transcription PCR (qRT-PCR)

The M-MuLV–RH First-Strand cDNA Synthesis Kit (BioLabMix, Novosibirsk, Russia) was used for single-stranded cDNA synthesis from the RNA template. Quantitative PCR was performed using the SYNTOL SYBR Green I+ROX kit (Syntol, Moscow, Russia) on the CFX96 Touch Real-Time PCR Detection System (BioRad Laboratories, Moscow, Russia). PCR was made in 15 µL of the reaction mixture under the following conditions: 1 cycle at 50 °C for 10 min; 1 cycle at 95 °C for 5 min; 40 cycles at 95 °C for 10 s and 60 °C for 30 s. PCR product melting curves were constructed under the following conditions: 10 s at 95 °C; 5 s at 65 °C; and 5 s at 95 °C. Nine genes with different expression patterns were chosen randomly for the qRT-PCR verification of the RNA-seq data. The primer design for determining the relative level of gene expression was made using the IDT PrimerQuest software (http://eu.idtdna.com/PrimerQuest/Home, accessed on 1 December 2023). The list of the primers used in study is presented in Table 2. To standardize the amount of cDNA template, qPCR was performed with the primers for the housekeeping gene *VuUBQ10* (*Vigun07g244400*) 5′-gtctaaggggaggaatgcagat-3′ and 5′-caaagatcaacctctgctggtc-3′ [49]. Each sample was amplified in three technical replicates. The differences among the genotypes were tested by a *t*-test, taking *p* ≤ 0.001 and *p* ≤ 0.05 as significant.

## 5. Conclusions

RNA-seq analysis performed in the current study revealed the DEG responses of different cowpea genotypes to high RH. Most of the DEGs are involved in response to light, external, and biotic stimuli, and plastid–thylakoid component-associated and hormone-related genes. Our results provide the next step toward identifying the master regulators controlling cowpea reactions to high RH, and also open new perspectives for understanding the complex regulatory mechanisms of responses to high humidity. We supposed that the DEGs associated with jasmonic acid signaling are key contributors in the maintenance of compact architecture under humid conditions.

Due to climate change, the selection of cowpea accessions adapted for cultivation in different regions is a very important task. New cultivars will be especially valuable in regions with high humidity, namely the Far East region in Russia. The identification of the molecular mechanisms responsible for some cowpea accessions that have a stable plant architecture and do not change their growth habit in high humidity conditions is very important for successful cowpea breeding. The identification of the molecular mechanisms due to which some cowpea accessions have a stable plant architecture and do not change growth habit type in high humidity conditions is very significant. In addition, it is necessary to continue the search of cowpea accessions with such a stable architecture. In summary, it allows for developing new approaches to directed breeding programs to expand the cowpea cultivation area beyond areas with an arid and semi-arid climate.

## Figures and Tables

**Table 1:** The log_2_(FC) values and *p*-values predicted by the RNA-seq data.

Gene	log_2_ (FC)	*p*-Value (RNA-Seq)
*Vigun01g020000*	9.40	3.27 × 10^−92^
*Vigun02g200300*	−6.08	1.61 × 10^−13^
*Vigun04g129200*	8.26	1.89 × 10^−36^
*Vigun05g198900*	8.18	4.61 × 10^−73^
*Vigun07g270500*	−7.32	3.02 × 10^−74^
*Vigun08g151600*	9.42	1.59 × 10^−125^
*Vigun08g216300*	7.16	6.73 × 10^−24^
*Vigun11g007900*	7.88	1.13 × 10^−62^
*Vigun11g015600*	7.02	7.05 × 10^−73^

**Table 2:** Primers used in the qRT-PCR verification of RNA-seq data.

Gene ID	Primer	Sequence (5′-3′)	Amplicon Length
*Vigun01g020000*	Forward	ACTCAGGTTCAAAGGATTTCCC	149
	Reverse	CTGTCCAATGACCATCTCAAG	
*Vigun02g200300*	Forward	GAGCAAAGGAGAAGTGGGTG	136
	Reverse	AAGAAAGCTCAACTCTGGACC	
*Vigun04g129200*	Forward	CACTGACCCAATCTACCCG	150
	Reverse	GTGACAATGGCTTGAACGAAG	
*Vigun05g198900*	Forward	TGGGACACTGCTGGTTTATCTG	141
	Reverse	GTCCACTCTCAACCACTTCTC	
*Vigun07g270500*	Forward	GACCACTCCTGACAAACCAA	139
	Reverse	TCTGCCTCCGTCAATTATCAC	
*Vigun08g151600*	Forward	CGGTTGTGGATTCGCTATTGC	136
	Reverse	TTCAGTTGGAAGAGGGAAAGG	
*Vigun08g216300*	Forward	TCAATGAGTTCGTCCGCAAG	131
	Reverse	CTCAGAGAATGGACCCAAGTAC	
*Vigun11g007900*	Forward	AGGACGTAGAAAAGCAAGGTG	148
	Reverse	TCTTTGGAAGACCACAACTCAG	
*Vigun11g015600*	Forward	TCTTGGACCCTTTTGAGCAG	143
	Reverse	AAACAGGGCATCTCGGAAG	

## Data Availability

Data is contained within the article and Supplementary Materials.

## Supplementary Materials

The following supporting information can be downloaded at: https://www.mdpi.com/article/10.3390/ijms252011056/s1.
