# Supplementary material for: Contrast Relative Humidity Response of Diverse Cowpea (Vigna unguiculata (L.) Walp.) Genotypes: Deep Study Using RNAseq Approach
Source: Int J Mol Sci. 2024 Oct 15;25(20):11056. doi: 10.3390/ijms252011056 (PMC11507454; doi:10.3390/ijms252011056)
Supplement: Supplementary file 1 [file ijms-25-11056-s001.zip › Figure_S1.pdf]

Accession / Location of reproduction	k-6	k-642	k-1783	k2056 (Lyanchihe)
AOS 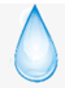	121,1±11,3 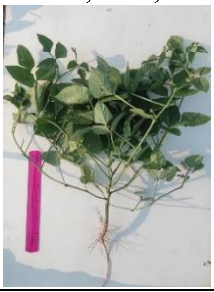	104,0±4,2 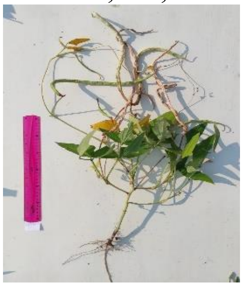	72,7±3,8 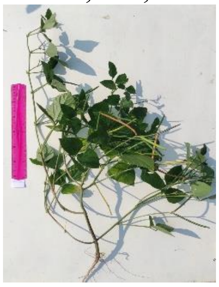	29,0±2,5 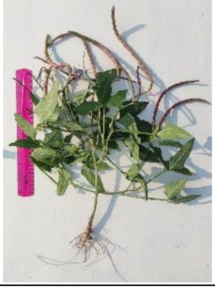
AdOS 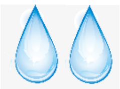	155,4±19,4 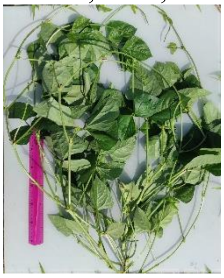	144,6±16,5 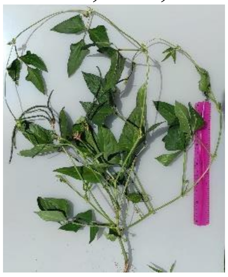	116,4±15,4 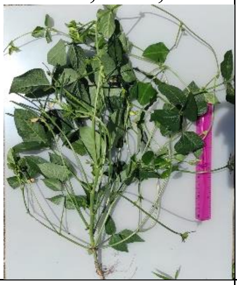	59,8±12,7 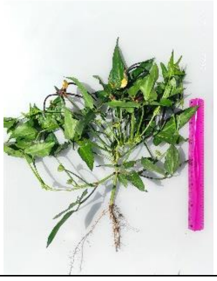
DVOS 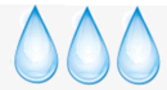	161,3±14,3 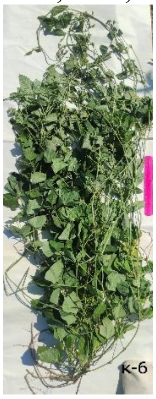	219,0±9,5 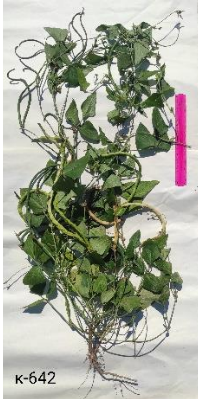	132,2±12,0 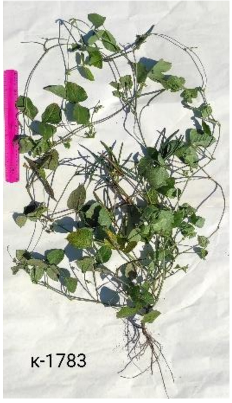	64,8±7,4 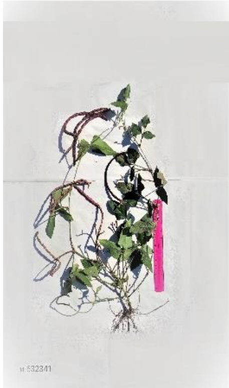

Figure_S1. Cowpea accessions in different ecological and geographical conditions at VIR experimental stations [12]. Reproduction was in three research locations with contrast RH conditions - in Astrakhan Province (AOS, sharply continental climate), Krasnodar Territory (AdOS, subtropical climate) and Primorye Territory (DVOS, monsoon climate). These regions are suitable for successful cowpea reproduction. The average values of plant length ± the standard error of the average (for all years of study) are shown above the photos of the accessions. Significant influence of the factor complex (genotype and research location) on the variability of morphological and phenological traits was detected. It was shown that phenological traits (number of days from sowing to emergence, to flowering and to pod maturity) and the length of internode and leaflets were dependent on research location. Variability range of the most studied traits was dependent on genotype. Stem length variation was dependent on research location but genotype had a stronger influence. In addition, the only stem length was correlated with sum of precipitation and RH. Excessive humidity had minimal influence on plants of cultivar "Lyanchihe" (it was observed not significant shoot elongation compared to other accessions).
