# Supplementary material for: Contrast Relative Humidity Response of Diverse Cowpea (Vigna unguiculata (L.) Walp.) Genotypes: Deep Study Using RNAseq Approach
Source: Int J Mol Sci. 2024 Oct 15;25(20):11056. doi: 10.3390/ijms252011056 (PMC11507454; doi:10.3390/ijms252011056)
Supplement: Supplementary file 1 [file ijms-25-11056-s001.zip › Figure_S2.pdf]

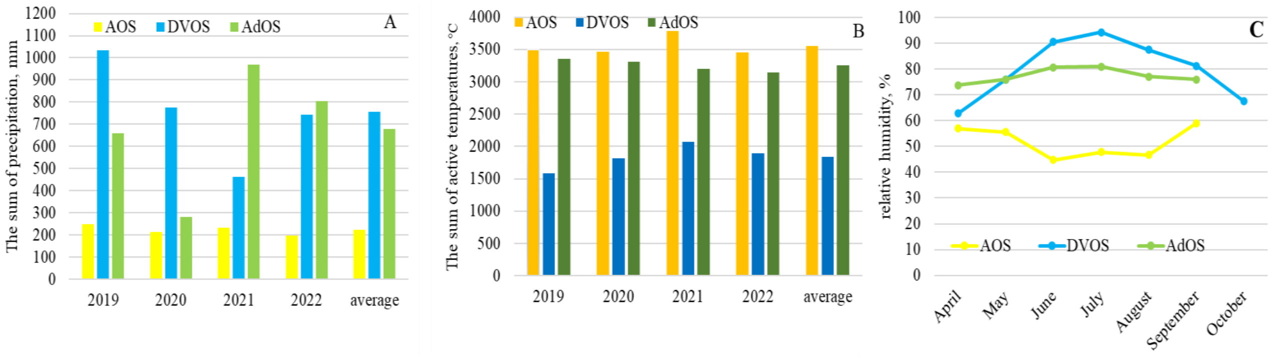

Figure_S2. Sum of the precipitation (A), the sum of active temperatures (B) and relative humidity (C) at VIR experimental stations during 2019-2022 years (in Astrakhan Province (AOS, sharply continental climate), Krasnodar Territory (AdOS, subtropical climate) and Primorye Territory (DVOS, monsoon climate)) [12].
